# The Effects of Walking or Walking-with-Poles Training on Tissue Oxygenation in Patients with Peripheral Arterial Disease

**DOI:** 10.1155/2012/985025

**Published:** 2012-09-25

**Authors:** Eileen G. Collins, Conor McBurney, Jolene Butler, Christine Jelinek, Susan O'Connell, Cynthia Fritschi, Domenic Reda

**Affiliations:** ^1^Department of Veterans Affairs, Center for Management of Complex Chronic Care, Edward Hines Jr., VA Hospital, Hines, IL 60141, USA; ^2^Department of Biobehavioral Health Science, College of Nursing, University of Illinois at Chicago, Chicago, IL 60612, USA; ^3^Department of Veterans Affairs, Research & Development, Edward Hines Jr., VA Hospital, Hines, IL 60141, USA; ^4^Cooperative Studies Program, Department of Veterans Affairs, Edward Hines Jr., VA Hospital, Hines, IL 60141, USA

## Abstract

This randomized trial proposed to determine if there were differences in calf muscle StO_2_ parameters in patients before and after 12 weeks of a traditional walking or walking-with-poles exercise program. Data were collected on 85 patients who were randomized to a traditional walking program (*n* = 40) or walking-with-poles program (*n* = 45) of exercise training. Patients walked for 3 times weekly for 12 weeks. Seventy-one patients completed both the baseline and the 12-week follow-up progressive treadmill tests (*n* = 36 traditional walking and *n* = 35 walking-with-poles). Using the near-infrared spectroscopy measures, StO_2_ was measured prior to, during, and after exercise. At baseline, calf muscle oxygenation decreased from 56 ± 17% prior to the treadmill test to 16 ± 18% at peak exercise. The time elapsed prior to reaching nadir StO_2_ values increased more in the traditional walking group when compared to the walking-with-poles group. Likewise, absolute walking time increased more in the traditional walking group than in the walking-with-poles group. Tissue oxygenation decline during treadmill testing was less for patients assigned to a 12-week traditional walking program when compared to those assigned to a 12-week walking-with-poles program. In conclusion, the 12-week traditional walking program was superior to walking-with-poles in improving tissue deoxygenation in patients with PAD.

## 1. Introduction

 Walking ability is impaired in patients with peripheral arterial disease (PAD) due to decreased blood flow to the skeletal muscle tissue used during walking. It has been demonstrated that walking distance is associated with the degree of impairment in the affected leg as measured by the ankle-brachial index (ABI) and the time to onset of claudication pain [1–6]. Likewise, it has been shown that walking exercise in patients with PAD prolongs the onset of claudication pain thus allowing the patient to walk longer but does not change the ABI [7–9].

 Recently, several investigators have used near-infrared spectroscopy (NIRS) to dynamically study the effect of walking on oxygen desaturation of the gastrocnemius muscle of the impaired leg during exercise [2, 10–14]. Investigators noted a marked decline in skeletal muscle tissue oxygenation (StO_2_) during walking exercise [2, 10–14]. Gardner et al. also identified that the rate of decline in calf muscle StO_2_ was significantly associated with initial claudication time and absolute claudication time in patients with PAD [2]. Three of the above studies examined the changes in tissue oxygenation, using NIRS, in patients with PAD before and after an exercise training program [12–14]. Figoni et al. [12] used a pretest-post-test design and reported greater exercise duration and lower mean exercise StO_2_ in 15 patients after a 3-month treadmill and calf exercise training program. Manfredini et al. [13] studied 55 patients with PAD who were given the option to participate in a structured or unstructured home exercise program for 34 ± 2 weeks. At the end of the study, those patients in the structured exercise program had an increased oxyhemoglobin area under the curve when compared to baseline; similar changes were not identified in the unstructured exercise group. Lastly, Tew et al. [11] studied calf-tissue oxygenation and treadmill walking response time after randomization to 12 weeks of arm-crank ergometry or a control group in 57 patients with intermittent claudication. Investigators identified that walking distance and time to nadir StO_2_ increased in those assigned to arm-cranking and there were no changes in the control group. No studies however compared leg muscle responses after a traditional walking program compared to a walking-with-poles program of rehabilitation in patients with claudication. 

The purpose of this study was to determine if there were differences in calf muscle StO_2_ parameters before and after 12 weeks of a traditional walking program versus a walking-with-poles exercise program. We reasoned that by using the poles to offload the legs, patients would have less claudication pain and be able to walk longer distance thus achieving a greater training effect. We hypothesized that the calf muscle tissue deoxygenation would be less in both groups after 12 weeks of exercise training than at baseline and that the decrease would be greater in those patients assigned to the walking-with-poles group. These data represent a subanalysis of data from a larger clinical trial comparing traditional walking to walking with poles [14].

## 2. Methods

### 2.1. Subjects

Patients with PAD were screened and recruited to participate in a randomized clinical trial designed to compare the effects of a traditional walking training program versus walking-with-poles exercise training program. A total of 2296 patients were screened for eligibility, 146 enrolled in the study [14]. Patients were recruited using radio and newspaper advertising, posted fliers, and letters of invitation sent to patients at the university and VA hospitals. For the purposes of this analysis, we included only those participants with an ABI of ≤0.90 (*n* = 91 patients). Of those 91 patients, 3 did not have StO_2_ analyses on their baseline progressive treadmill test due to equipment malfunction and 3 had questionable data resulting in a final sample of 85 patients. Of those 85 patients, 45 were assigned to the walking-with-poles group and 40 patients were assigned to the traditional walking group. 

### 2.2. Research Design

The major study was a randomized clinical trial to examine the effects of a traditional walking program compared to walking with poles on walking endurance and perceived physical function in patients with PAD. This subanalysis provides an in-depth examination of StO_2_ responses to exercise training. Our primary outcome focused on changes the patients realized walking on the constant work rate treadmill test whereas this analysis used data from the progressive treadmill test. At two minutes on the constant work rate test, the speed and incline increased to 85% of the patient's workload on the progressive treadmill test resulting in a sudden decline in StO_2_. Due to the gentle nature of the progressive treadmill protocol used, we were able to detect more subtle changes in StO_2_ and onset of claudication pain. 

 The intervention consisted of traditional walking or walking with poles three times weekly. Patients were randomized using computer-generated permuted blocks and group assignment was managed by the study statistician. The training programs for both groups were identical except that one group exercised with poles and the other group did not. Patients assigned to the walking-with-poles group received training on the use of the poles prior to beginning the exercise program. Additionally, patients were coached on proper poling mechanics during the training period if needed. The training program incorporated interval training whereby exercise consisted mostly of low-to-moderate intensity training at the start of the program and progressed to moderate-to-high intensity. Exercise intensity was determined by heart rate associated with a percentage of oxygen uptake obtained during the metabolic exercise treadmill tests. Exercise duration and intensity was adjusted every three weeks. During the first weeks, patients walked for 30 minutes (20% light intensity, 60% moderate intensity, and 20% hard intensity). By weeks 10–12, patients walked for 55 minutes (10% light intensity, 45% moderate intensity, and 45% hard intensity). Light intensity was defined as 25–44% peak VO_2_, moderate intensity was 45–59% peak VO_2_, and hard intensity was 60–84% VO_2_ peak. The program was designed so that patients walked on the treadmill two days/week and outdoors or in the hospital corridors one day/week. The exercise interval was adjusted based on the patient's pain tolerance. For example, some patients could only walk for one to two minutes at the higher intensities. In order for them to achieve 20% total time at the harder intensities, multiple short intervals were completed. Exercise intervals were gradually lengthened and rest times between intervals were gradually decreased.

### 2.3. Measurements

Demographic information, height, weight, comorbid conditions, and medication history were obtained from the patient and reviewed in the patient medical record. A physical exam was completed prior to participation in the study.

#### 2.3.1. Ankle-Brachial Index

The ABI was measured in the more severely affected leg at baseline and 12 weeks after training. The dorsalis pedis or posterior tibial arteries were used for measurement. The location of the signal was recorded and used consistently throughout the study for that individual subject. After the patient rested comfortably in a supine position for 15 minutes, Doppler ultrasound was used to measure the systolic pressure in the right and left arms and in the ankle of the most severely affected leg. The arm with the highest systolic pressure was used to calculate the ABI [15].

#### 2.3.2. Symptom-Limited Treadmill Test Protocol

Patients were tested using a gentle treadmill protocol that was developed for patients with PAD [6, 16]. Increases in percent grade occurred every 30 seconds, and, after the first 6 minutes, speed increased every 3 minutes. Validity and reliability of the treadmill protocol has been previously reported [17]. Electrocardiogram and StO_2_ of the gastrocnemius muscle were monitored continuously throughout the test. Initial claudication time was defined as the time during the treadmill test when the subject experienced pain in the affected leg. The absolute walking time was defined as the time walked by the patient on the progressive treadmill test.

#### 2.3.3. Metabolic Values

Peak oxygen uptake was measured using the MedGraphics CPX/D System (Medical Graphics Corp, St. Paul, MN, USA). The metabolic cart was calibrated using a 3 L syringe prior to each test and the analyzers were calibrated using references gasses and room air. Patients breathed through a mouthpiece and their nose was clipped with a standard nose clip. Oxygen uptake was averaged over 30 s and the highest value at peak exercise was recorded.

#### 2.3.4. Near-Infrared Spectroscopy Measures (NIRS)

Skeletal muscle tissue oxygenation was measured prior to, during, and after exercise using the NIRS spectrometer (InSpectra 325, Hutchinson Technology, Inc., Hutchinson, MN, USA). A 25 mm probe attached to an optical cable was used to noninvasively measure the percentage of hemoglobin oxygen saturation of the tissue beneath the near-infrared light. The InSpectra machine was calibrated prior to each test according to known high and low reference standards [18]. The NIRS probe was attached to the medial section of the gastrocnemius muscle of the patient's most severely affected leg. Calf circumference measurements were taken to assure consistent placement of the probe. The probe was placed into a specially designed adhesive housing unit to reduce ambient light. The probe was then secured to the leg to avoid excessive motion and possible tripping. The StO_2_ measures were recorded every 3.5 seconds using the InSpectra software. The StO_2_ values used in this analysis were the StO_2_ value recorded after 2 minutes of standing prior to exercise (StO_2_ rest), time walked when the patient reached the nadir StO_2_ value (time to nadir StO_2_ value), StO_2_ value at peak exercise (StO_2_ peak), and absolute and relative drop in StO_2_ (absolute drop in StO_2_ = rest StO_2_ − minimal StO_2_ value, relative drop in StO_2_ = [rest StO_2_ − nadir StO_2_ value]/rest StO_2_).

#### 2.3.5. Ratings of Perceived Pain

Ratings of perceived leg pain were obtained every minute using the Borg ratio scale [19]. Patients were instructed to notify the staff at the onset of leg pain. The initial claudication time is the time that patients either notified the staff of the onset of pain or the time they first reported pain when asked every minute. 

### 2.4. Statistical Analysis

Measures of central tendency were completed to describe the sample. The two groups were compared at baseline using the *t*-test for independent samples and Mann Whitney *U* test. Data that were not normally distributed were log transformed. Regression analyses were used to compute the initial decline in slopes for StO_2_ values (StO_2_ value regressed over time). Pearson r correlations were completed to examine relationships between the variables. Paired *t*-tests were used to describe changes in measures from baseline to post-12 weeks of training. Independent *t*-tests were used to examine differences between groups. Intent-to-treat analyses using the baseline value carried forward were completed for the comparisons between groups. The criterion for significance was *P* < .05. 

## 3. Results

The sample consisted of primarily older (age = 69.4 ± 9.2 years) men. The ABI was .63 ± .19. Over one-third of the patients were current smokers, half were diabetic, and the majority of patients received treatment for hypertension and dyslipidemia (Table 1). Patients were generally unfit with a peak oxygen uptake of 14.3 ± 2.9 mL · kg^−1^ · min⁡^−1^. The walking-with-poles group was older than the traditional walking group. No other differences in baseline demographic characteristics were identified. 

 At baseline, calf muscle oxygenation decreased from 56 ± 17% prior to the treadmill test to 16 ± 18% at peak exercise (71% relative decline from rest to peak exercise). Patients walked 4.45 ± 3.67 minutes before experiencing claudication pain and 4.39 ± 3.99 minutes when the StO_2_ reached its nadir value. There was no difference between the time elapsed when the StO_2_ reached its nadir value and pain onset (*P* = .87). Although all patients reported claudication pain, exercise was not limited by claudication pain for all patients. Therefore, we examined the onset of claudication pain and time to nadir StO_2_ for those whose exercise was limited by claudication pain only (*n* = 72). Patients walked 3.35 ± 2.57 minutes before experiencing claudication pain and 3.67 ± 3.49 minutes when the StO_2_ reached its nadir value. There was no difference between the time elapsed when the StO_2_ reached its nadir value and pain onset (*P* = .40). After exercise, the time for the StO_2_ to return to half of its resting value was 1.3 ± 1.3 min and 2.2 ± 2.1 minutes to return to resting values. The initial claudication time and absolute walking time were not associated with the StO_2_ values at rest (Table 2) but were moderately related to the time elapsed prior to reaching the nadir StO_2_ value (*r* = .42, *P* < .001; *r* = .58, *P* = .001, resp.). There was also a small to moderate relationship between onset of claudication pain and the exercise slope decline in StO_2_ (*r* = .28, *P* = .011). Relationships were similar between initial claudication time and absolute walking time and the time elapsed prior to reaching the nadir StO_2_ values when the analysis was restricted to those whose exercise was limited by claudication (*r* = 48, *P* ≤ .001 and *r* = .47, *P* < .001).

Data comparing groups after exercise training were analyzed using intent-to-treat analysis for all patients. The change in time elapsed prior to reaching nadir StO_2_ values between baseline and 12 weeks was related to the change in absolute walking time between baseline and 12 weeks (*r* = .65, *P* < .001) and initial claudication time at 12 weeks (*r* = .33, *P* = .002). Analysis of covariance was completed to compare the groups with age as the covariate since there was a difference in age at baseline (Table 3). The change in the time elapsed prior to reaching nadir StO_2_ values (*P* = .002) and absolute walking time (*P* = .002) were greater in the traditional walking group when compared to the walking-with-poles group. There was, however, no difference in the change in initial claudication time between the two groups (*P* = .38). 

**Figure 1:**
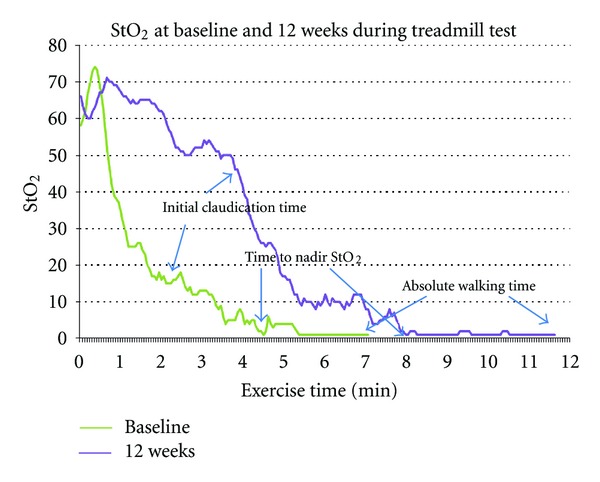
StO_2_ data from sample patient at baseline and 12 weeks.

When the analyses were restricted to those who completed the exercise training only (*n* = 71), the change in time elapsed prior to reaching nadir StO_2_ values between baseline and 12 weeks was related to the change in absolute walking time between baseline and 12 weeks (*r* = .57, *P* < .001) and initial claudication time at 12 weeks (*r* = .32, *P* = .004). Data from a sample patient are depicted in Figure 1. Recovery time was unchanged when baseline and 12-week values were compared (baseline = 2.45 ± 2.17 min, 12 wk = 2.19 ± 1.94 min, *P* = .81). After 12 weeks of exercise training, within-group changes in initial claudication time, absolute claudication time and time elapsed prior to reaching nadir StO_2_ values increased (Table 4.) The time elapsed prior to reaching nadir StO_2_ values increased more in the traditional walking group when compared to the walking-with-poles group (walking group = 4.01 ± 5.13 minutes, walking-with-poles group = .89 ± 3.56 minutes, *P* = .009). Similarly, absolute walking time increased more in the traditional walking group than in the walking-with-poles group (walking group = 3.97 ± 3.14 minutes, walking-with-poles group = 2.24 ± 2.41 minutes, *P* = .017). There was no difference between the two groups in initial claudication time (walking group = 1.16 ± 3.64 minutes, walking-with-poles group = .85 ± 3.46 minutes, *P* = .69). Since there was a significant difference in age between the two groups, age was entered as a covariate yet it did not contribute significantly to the model. No differences in recovery time were noted between the traditional walking and walking-with-poles groups at the 12-week follow-up time period (traditional walking recovery time = 1.26 ± 1.43 min, walking with poles = 1.41 ± 1.76 min, *P* = .70). No significant differences were identified in peak oxygen consumption or ABI within or between the two groups from baseline to 12 weeks using intent-to-treat analysis or restricting the analysis to those who completed the training only.

## 4. Discussion

 There are three major findings of this study: (1) the time elapsed before reaching nadir StO_2_ values is associated with initial claudication time and absolute walking time, (2) there was no difference in the time associated with the nadir of StO_2_ and the onset of claudication pain, and (3) the time to nadir StO_2_ of the gastrocnemius muscle was greater after 12 weeks of exercise training in the traditional walking group when compared to the walking with poles group. 

 As Bauer notes, during higher work rates and apparent blood flow limitation, muscle oxygen desaturation becomes more rapid in PAD subjects [19]. Thus, one would expect that oxygen desaturation would be associated with onset of claudication and walking duration. Our findings reflect these assumptions and were similar to those reported by Gardner et al. [2], that is, shorter time to reach nadir StO_2_ values were associated with shorter initial claudication time and total walking duration. Second, in Gardner's study, relationships between initial claudication time (*r* = .339), absolute walking time (*r* = .68) and measured time to minimal exercise StO_2_ were similar to our relationships between initial claudication time (*r* = .42), absolute walking time (*r* = .58) and measured distance to nadir StO_2_. These findings suggest that our sample patients were similar to patients in other studies [2, 19].

 Exercise-associated decline in StO_2_ of the gastrocnemius muscle was less after 12 weeks of exercise training when compared to baseline. Wang and colleagues recently demonstrated in 17 subjects with PAD who completed a 24-week walking program that an increase in the number of capillaries in contact with type IIx and IIa calf muscle fibers was related to an improved pain-free walking time (*r* = .69 and *r* = .62, *P* < .05) [8]. Investigators hypothesized that the ratio of capillaries to muscle fibers affects oxygen supply thus delaying the mismatch between supply and demand [8]. Figoni et al. [12] and Manfredini et al. [13] both report greater exercise StO_2_ area under the curve postexercise training when compared to preexercise training. In our study, an increase in the time to nadir StO_2_ values was associated with delayed initial claudication time and improved absolute walking time. Our findings provide further evidence that walking exercise improves tissue muscle oxygenation in patients with PAD. Likewise, our findings support the possibility that the onset of claudication and walking duration are linked by the decline in tissue oxygenation. The onset of claudication and the time elapsed prior to reaching the nadir StO_2_ values were nearly identical. Thus, the patient's perception of pain is a good indicator of StO_2_ in the calf muscle.

Exercise-induced decline in gastrocnemius muscle StO_2_ was less in patients assigned to the traditional walking program than in patients assigned to the walking-with-poles exercise training program. We had originally hypothesized that a walking training program with poles for patients would be advantageous because the poles would “offload” the legs during periods of claudication allowing additional walking exercise. Indeed, Oakley et al. [20] investigated the immediate effects of walking with Nordic poles on 20 patients with PAD and found that claudication distance increased from 77 m without the poles to 130 m with the poles (*P* < .0001) and the level of claudication pain also decreased (*P* = .0002). Our group has also previously reported that patients had decreased calf pain when walking with poles. Oakley et al. [20] compared muscle histology of 36 patients randomly assigned to 12 weeks of treadmill training, strength training or control. After the 12 weeks, only the treadmill group had histological changes in the muscle. The increase in muscle ischemia with treadmill training was associated with a marked increase in muscle damage. Hiatt suggests that skeletal muscle dysfunction and associated muscle metabolic state can be ameliorated with treadmill exercise training. Tew et al. [11] provides evidence that even when the exercising muscle is not the calf muscle, tissue oxygenation can improve. Our evidence is equivocal. Clearly, the patients assigned to the traditional walking group experienced a prolonged exercise time before experiencing claudication pain, prolonged their absolute walking time and increased the amount of time exercising until reaching nadir values. In the walking-with-poles group, there were no changes over time when an intent-to-treat approach was used. However, when the analysis was restricted to those who completed the training program only, there was a difference in absolute walking time and a trend toward increasing time to initial claudication pain onset as well as nadir StO_2_. The explanation for these findings may be that leg metabolic changes in the calf muscle occurred to a greater extent in those assigned to the walking-with-poles group or we may not have had enough statistical power to detect changes that were present.

Another explanation for this finding may be that the intensity of our training program was based on oxygen uptake associated with heart rate response to exercise. With the addition of the upper body to the exercise regime for patients assigned to the walking-with-poles group, exercise intensity was augmented using the upper body. Thus, the work accomplished by the leg muscles may have been less even though overall exercise intensity was consistent between the two groups of patients. Both groups trained on the treadmill and outdoors. Patients assigned to the walking with poles group were unable to use the handrails on the treadmill for balance; added anxiety may have increased the cardiovascular response thus keeping the overall work accomplished lower in some patients. 

Our study had several limitations that may limit the generalizability of the findings. First, although age was not a contributing factor to the differences between the groups, the walking-with-poles group was older than the traditional walking group. We cannot exclude that the differences in exercise tolerance and willingness to push limits may differ between someone who is 67 years old (mean age for walking group) and someone who is 72 years old (mean age for walking-with-poles group). This five-year age difference may have influenced our outcomes in ways that we did not measure. Next, the sample was primarily men and thus cannot be generalized to women. Additionally, in order to include only those with compressible vessels, we excluded 17 patients who were initially randomized to our study. Although this limited the variability, it also reduced power in our statistical testing. Lastly, although all of our patients experienced claudication pain, not all patients stopped exercise due to claudication pain. Including patients that did not stop exercise due to claudication pain may have affected the study results.

Additional studies examining different walking intensities in patients with peripheral arterial disease should be tested. Further, it may be that use of walking poles early in the rehabilitative effort may augment traditional walking training later by assisting patients to train longer early on. Further research in this area is warranted.

## 5. Conclusion

In summary, we conclude that there is a significant decline in StO_2_ in the gastrocnemius muscle during walking exercise in patients with peripheral arterial disease reflecting muscle tissue ischemia. Contrary to our hypothesis, after 12 weeks of exercise training, absolute walking time and time to reach nadir muscle tissue oxygenation were prolonged in the patients assigned to the traditional walking program more than those assigned to a walking-with-poles exercise training program reflecting greater tissue perfusion and enhancement of arteriovenous oxygen extraction. The precise mechanism for these differences remains unknown but these findings may provide some insight to tissue adaptations to exercise stimulus and may therefore be useful to PAD rehabilitation programs.

## Figures and Tables

**Table 1:** Demographic characteristics of the sample.

Characteristic	Combined	Walking with poles	Traditional walking	*P* value
	*n* = 85	*n* = 45	*n* = 40	
Age, years	69.4 ± 9.1	71.7 ± 9.2	66.8 ± 8.5	.012
ABI	0.63 ± 0.18	0.62 ± 0.20	0.63 ± 0.17	.75
Weight, kg	89.6 ± 16.4	89.1 ± 14.4	90.4 ± 18.7	.71
BMI, kg/m^2^	29.0 ± 4.9	29.2 ± 4.4	28.9 ± 5.5	.77
ICT, min	4.45 ± 3.67	4.18 ± 3.34	4.70 ± 3.98	.32
AWT, min	8.47 ± 4.05	8.58 ± 3.92	8.35 ± 4.22	.77
Peak oxygen uptake, ml·kg^−1^·min^−1^	14.3 ± 2.9	14.0 ± 2.7	14.6 ± 3.1	.39
Sex, % male	93%	91%	95%	.66
Race, % caucasian	78%	80%	77%	.78
Current smoking, % yes	35%	36%	33%	.66
Diabetes,% yes	49%	47%	52%	.93
Hypertensive, % yes	85%	78%	92%	.54
Dyslipidemia, % yes	74%	76%	73%	.79

AWT: absolute walking time; ICT: initial claudication time.

**Table 2:** Relationships between selected variables and initial claudication and absolute walking times (*n* = 85).

Variables	ICT Pearson *r *	*P* value	AWT Pearson *r *	*P* value
StO_2_ at rest	.07	.51	.02	.87
Peak exercise StO_2_	.14	.22	.23	.04
Absolute decline in StO_2_ during exercise	−.19	.08	−.12	.30
Relative decline in StO_2_ during exercise	−.27	.01	−.17	.14
Time walked to nadir StO_2_ value	.42	.000	.58	.000
Recovery time to baseline StO_2_	−.31	.02	.26	.04
Recovery StO_2_ at 1 min	.37	.001	.31	.01
Recovery StO_2_ at 3 min	.36	.001	.37	.001
Recovery StO_2_ at 5 min	.28	.01	.36	.001
Exercise slope	.28	.01	.18	.11
Recovery slope	.18	.11	.21	.07

AWT: absolute walking time; ICT: initial claudication time.

**Table 3:** Exercise times at baseline and 12 weeks, intent-to-treat analysis (*n* = 85).

	Traditional walking group		Walking-with-poles group		
	Walking time minutes (mean ± SD) *n* = 40	Within group *P* value	Walking time minutes (mean ± SD) *n* = 45	Within group *P* value	Between group *P* value
Initial claudication time					
(i) Baseline	3.95 ± 2.97	.034	3.33 ± 2.75	.15	
(ii) 12 wk	5.23 ± 4.29		3.98 ± 3.50		
(iii) Change	1.28 ± 3.68		0.64 ± 2.94		.38
Absolute walking time					
(i) Baseline	8.01 ± 4.01	.000	7.76 ± 4.01	.92	
(ii) 12 wk	11.30 ± 5.14		7.83 ± 5.57		
(iii) Change	3.73 ± 3.15		1.74 ± 2.42		.002
Time to nadir value					
(i) Baseline	4.25 ± 4.16	.000	3.69 ± 3.07	.22	
(ii) 12 wk	7.94 ± 5.89		4.30 ± 3.87		
(iii) Change	3.59 ± 5.19		0.60 ± 3.23		.002

Time to nadir value: time elapsed to reach nadir StO_2_ value. Above analyses were adjusted for age.

**Table 4:** Exercise times at baseline and 12 weeks for those who completed training (*n* = 71).

	Traditional walking group		Walking-with-poles group		
	Walking time minutes (mean ± SD) *n* = 36	Within group *P* value	Walking time minutes (mean ± SD) *n* = 35	Within group *P *value	Between group *P* value
Initial claudication time					
(i) Baseline	4.69 ± 3.98	.043	4.18 ± 3.34	.135	
(ii) 12 wk	5.86 ± 4.80		5.03 ± 4.11		
(iii) Change	1.16 ± 3.64		0.85 ± 3.46		.69
Absolute walking time					
(i) Baseline	8.35 ± 4.22	.000	8.60 ± 3.92	.000	
(ii) 12 wk	12.31 ± 4.27		10.83 ± 3.77		
(iii) Change	3.97 ± 3.14		2.24 ± 2.41		.017
Time to nadir value					
(i) Baseline	4.05 ± 4.15	.000	4.55 ± 3.70	.132	
(ii) 12 wk	8.07 ± 6.05		5.45 ± 4.45		
(iii) Change	4.01 ± 5.13		0.89 ± 3.56		.009

Time to nadir value: time elapsed to reach nadir StO_2_ value. Above analyses were adjusted for age.

## Abbreviations

ABI: Ankle-brachial index
AWT: Absolute walking time
ICT: Initial claudication time
NIRS: Near-infrared spectroscopy
PAD: Peripheral arterial disease
StO_2_: Skeletal muscle tissue oxygenation.
